# Impact of cardiac interoception cues and confidence on voluntary decisions to make or withhold action in an intentional inhibition task

**DOI:** 10.1038/s41598-020-60405-8

**Published:** 2020-03-06

**Authors:** Charlotte L. Rae, Aysha Ahmad, Dennis E. O. Larsson, Marta Silva, Cassandra D. Gould van Praag, Sarah N. Garfinkel, Hugo D. Critchley

**Affiliations:** 1 School of Psychology, University of Sussex, Brighton, UK; 2 Sackler Centre for Consciousness Science, University of Sussex, Brighton, UK; 3 Department of Neuroscience, Brighton & Sussex Medical School, Brighton, UK; 4 Department of Psychiatry, University of Oxford, Oxford, UK; 5 Sussex Partnership NHS Foundation Trust, Brighton, UK

**Keywords:** Cardiovascular biology, Human behaviour

## Abstract

Interoceptive signals concerning the internal physiological state of the body influence motivational feelings and action decisions. Cardiovascular arousal may facilitate inhibition to mitigate risks of impulsive actions. Baroreceptor discharge at ventricular systole underpins afferent signalling of cardiovascular arousal. In a modified Go/NoGo task, decisions to make or withhold actions on ‘Choose’ trials were not influenced by cardiac phase, nor individual differences in heart rate variability. However, cardiac interoceptive awareness and insight predicted how frequently participants chose to act, and their speed of action: Participants with better awareness and insight tended to withhold actions and respond slower, while those with poorer awareness and insight tended to execute actions and respond faster. Moreover, self-reported trait urgency correlated negatively with intentional inhibition rates. These findings suggest that lower insight into bodily signals is linked to urges to move the body, putatively by engendering noisier sensory input into motor decision processes eliciting reactive behaviour.

## Introduction

Intentional inhibition is the volitional withholding of cognitive processes, which include the withholding of motor action^1^, thoughts^2^, and memories^3^. In ‘what-when-whether’ models of voluntary action decisions^1,4^, intentional inhibition encompasses the ‘whether’ category of *whether* to perform an action or inhibit it. This can occur in response to an external decision cue, or to an internal thought or prompt^5^.

Within the brain, intentional decisions to withhold actions are associated with enhanced activation within prefrontal and motor preparation areas, including the pre-supplementary motor area (preSMA), extending ventrally to mid-cingulate cortex and the rostral cingulate zone, and the dorsolateral prefrontal cortex and inferior frontal gyrus^4,6–8^. Interestingly, neuroimaging studies of intentional inhibition also show activation of the anterior insular cortex^4,9^. The insula is implicated as a substrate for motivational feelings, through the integration of interoceptive information concerning the internal state of the body, cueing homeostatic adjustments of behaviour through midline motor pathways^10–12^. This implies that afferent information concerning bodily physiology can influence intentional inhibition decisions, for example via ‘somatic markers’, proposed by Damasio^13^ to function as rapid, unconscious, cues to guide behaviour.

Beyond interoceptive cues, signals from other sensory domains, such as vision, can guide intentional action decisions. A modified version of a Go/NoGo task incorporates ‘Choose’ trials that require participants to decide whether to act or to withhold a button press. In this task, subliminal (unconscious) priming by visual cues that precede Choose trials can influence motor choices, by reducing a participant’s tendency to withhold action^14^. Thus sensory cues which do not enter awareness may nevertheless shape volitional motor behaviour.

In the interoceptive domain, cardiovascular arousal, signalled by activation of baroreceptors in vessel walls with each heartbeat, influences many cognitive processes, including pain, visual perception of fearful stimuli, and memory^15^. Arterial baroreceptors are activated as blood is ejected out of the heart. The maximal activation of aortic and carotid baroreceptors typically peaks ~300 ms after an ECG R-wave^15–17^. In the domain of action control, we previously observed that reactive response inhibition, as measured from performance on the stop signal task, is influenced by such heartbeat signals^18^. Participants showed increased response inhibition efficiency (indexed by shorter stop signal reaction times) when ‘stop’ cues were presented at the point within the cardiac cycle when the heart contracts (systole), eliciting baroreceptor afferent firing, compared to when the heart fills between beats (diastole) and baroreceptors are quiescent. These momentary interoceptive cardiac cues are also associated with the timing of voluntary movements, with onset of actions to sample information more likely to coincide with systole^19,20^, and the offset of self-paced action sequences with diastole^21^. However, it is yet to be established if such cardiac cycle effects impact upon ‘whether’ voluntary decisions to make or withhold action.

In this study, we used a modified Go/NoGo task that incorporated ‘Choose’ trials on which participants chose whether to act or to withhold a button press. The task was combined with online dynamic monitoring of participants’ electrocardiograms (ECG), to deliver the onset of each trial at either cardiac systole or diastole. Given the facilitatory effect of systole on reactive response inhibition in the stop signal task^18^, and the long-held view that heartbeats may broadly invoke inhibitory processes across behavioural domains^22^, we predicted that heartbeats would promote intentional inhibition, such that participants would choose to withhold button presses more frequently during systole than diastole.

A previous literature describes the effects of action inhibition on changes to heart rate, with cardiac deceleration occurring during the inhibition of movements^23,24^, including intentional inhibition on the ‘marble’ task^25^. Here, we focused instead on the impact that momentary cardiac cues have on intentional inhibition, dynamically monitoring participants’ ECG such that trial onsets were delivered accurately even with fluctuations in heart rate.

Individual differences exist in interoceptive sensitivity, expressed across dissociable dimensions of interoception. These include objective interoceptive *accuracy* (according to performance on interoceptive tasks), subjective (trait) interoceptive *sensibility* (measured from self-report scales), and metacognitive interoceptive *awareness* or *insight* (reflecting trial-by-trial correspondence between subjective and objective measures)^26,27^. In addition, *trait interoceptive prediction error* reflects the magnitude of a discrepancy between an individual’s objective (*accuracy*) and belief-based (trait) subjective (*sensibility*) dimensions of interoception^28,29^. Such individual differences in psychological dimensions of interoceptive sensitivity can affect the impact of cardiac cues on cognition^30^. By extension, interoceptive abilities may influence voluntary decisions to make or withhold action.

In addition to measuring participants’ interoceptive abilities, we also investigated the relationship between reactive and intentional inhibition, as measured by NoGo and Choose-NoGo trials, with autonomic reactivity, measured by heart rate variability, and with trait impulsivity, measured by self-report questionnaires. Heart rate variability (HRV), which indexes cardiac (parasympathetic) vagal tone, is positively associated with enhanced executive function and emotion regulation^31^. Greater HRV predicts better response inhibition on the stop signal task^32^, and better suppression on an intentional thought inhibition paradigm^33^, suggesting that HRV may similarly be associated with volitional action inhibition. Moreover, an impulsive neurocognitive endophenotype may predispose individuals to poorer inhibitory control^34^, whereby higher trait impulsive individuals may choose to move more often, and choose to intentionally withhold actions less often, than individuals with lower trait impulsivity.

## Methods

### Participants

43 right-handed participants gave written informed consent to participate. The data failed to save for one participant, and one further participant was excluded from analysis due to a very large number of NoGo commission errors (>2 standard deviations from group mean). Data are presented from the remaining 41 participants (17 male, age 18–28, mean 22 years). Participants were recruited from students at the University of Sussex who had no reported history of psychiatric or neurological disorders, and no intake of medications affecting neural or peripheral physiological function. Prospective participants reporting colour-blindness were excluded. The study was approved by the Brighton and Sussex Medical School Research Governance and Ethics Committee, and all research was performed in accordance with relevant guidelines and regulations.

### Cardiac intentional inhibition task

**Figure 1:**
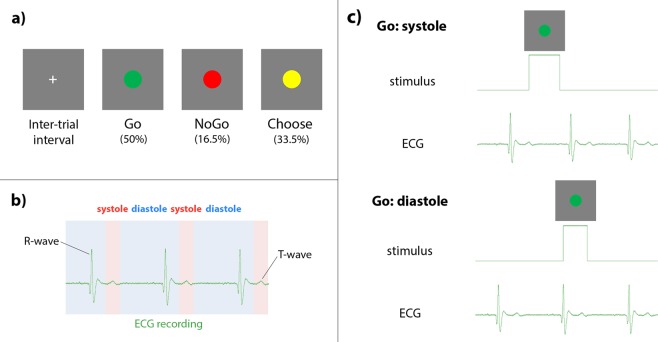
Cardiac intentional inhibition task. (**a**) Intentional inhibition task: following an inter-trial interval, on Go trials (50%) cues instructed participants to make a button press, on NoGo trials (16.5%) to withhold a button press, and on Choose trials (33.5%) to decide whether to execute or inhibit a button press. Stimuli enlarged for illustrative purposes. (**b**) Cardiac cycle in relation to ECG signal: systole (cardiac contraction) is signalled neurally by arterial baroreceptor firing. The activation of aortic and carotid baroreceptors is maximal 280 ms after the R-wave. (**c**) Example cardiac timing of task cue onsets. On systole trials, the onset of the task cue is 290 ms following R-wave; on diastole trials, the onset of the task cue is 10 ms prior to the R-wave.

Participants performed an intentional inhibition task in which the onset of the motor cues was timed to either cardiac systole (when the heart is contracting, and baroreceptors in arterial walls are activated) or cardiac diastole (when the heart is relaxed between beats, and baroreceptors are quiescent). The task was presented using Cogent2000 (version 1.32, http://www.vislab.ucl.ac.uk/cogent_2000.php) in Matlab (R2013a, Mathworks). Task stimuli comprised green, red, and yellow circles, presented in the centre of the screen on a grey background (Fig. 1a). Green Go cues indicated a button press of the left arrow key to be made using the right index finger. Red NoGo cues indicated the participant should withhold their button press. Yellow ‘Choose’ cues indicated participants should choose whether to press the button or to withhold. There were 400 trials in total: 200 Go trials (50%), 66 NoGo (16.5%), and 134 Choose (33.5%). The higher frequency of Go trials was designed to invoke a prepotent tendency to go, as in traditional Go/NoGo tasks, so that withholding on the NoGo trials was sufficiently challenging to invoke reactive inhibitory control^35–37^. 50% of each trial type were delivered at cardiac systole, and 50% at diastole. Participants were instructed to respond as quickly as possible on the Go trials, to withhold button presses on NoGo trials, and to choose quickly and make a fresh decision whether to press the button or not on each Choose trial. All cues were presented for a maximum duration of 1000 ms, with the trial ending sooner if the participant pressed the button. In between trials, a white fixation cross was displayed in the centre of the screen for a duration of 1000 ms, plus the duration during which the participants’ ECG was monitored to establish the time of the next R-wave peak (see ‘*ECG recording and stimulus delivery*’ below). The task was divided into four blocks of 100 trials, giving participants a rest break in between each block, in order to reduce movement during task performance and ensure that ECG recording was uncontaminated by movement artifact. The task took approximately half an hour to complete. An in-house Matlab script calculated four key indices of motor behaviour: 1) reaction times on Go trials, 2) % of NoGo trials on which participants made commission errors (pressed the button instead of withholding), 3) % of Choose trials on which participants decided to act (% Choose-Go), and 4) reaction times on Choose-Go trials.

### ECG recording and stimulus delivery

The onset of task cues was synchronised to specific time points within the cardiac cycle (Fig. 1b) using ECG recording, with Cambridge Electronic Design (CED) hardware and Spike2 physiological recording software (version 7.18), to interface cardiac events with the task in Matlab. Three Ag/AgCl electrodes (3 M Healthcare, Neuss, Germany) were attached with foam tape: two on the upper left and right chest, and a ground electrode above the left hipbone. The ECG signal was sampled at 1000 Hz, amplified (1902, CED) and relayed to Spike2 recording software via an analogue-to-digital recorder (1401, CED). An interactive threshold in the Spike2 recording isolated each ECG R-wave peak. This enabled the dynamic monitoring of inter-beat intervals for three beats prior to each trial, giving a median inter-beat interval to provide a precise temporal prediction of the next R-wave peak. Task cues could thus be delivered at either cardiac systole (290 ms following R-wave peak), or cardiac diastole (10 ms prior to R-wave peak) (Fig. 1c).

Although electrical depolarisation events, evident in the QRS complex of an ECG, are critical for initiating cardiac contraction, they do not correspond in time to the neural and cognitive perception of a heartbeat, which is signalled to the central nervous system (in part) via baroreceptor activation^15^. Imaging and catheter studies of cardiac function suggest that arterial baroreceptor discharge in the aorta and carotid sinus peaks around 300 ms after the ECG R-wave. The pre-ejection period between the start of myocardial depolarisation (QRS ECG complex) and the triggered onset of ventricular contraction is typically 130–160 ms^16,38^. The following left ventricular ejection time (LVET) is ~350–500 ms, with peak systolic pressure occurring within 100 ms of LVET^16,39,40^. The delay between left ventricular and systolic pressure peaks is ~50 ms^17^, altogether suggesting the activation of aortic and carotid baroreceptors is maximal after 280 ms from the R-wave.

**Figure 2:**
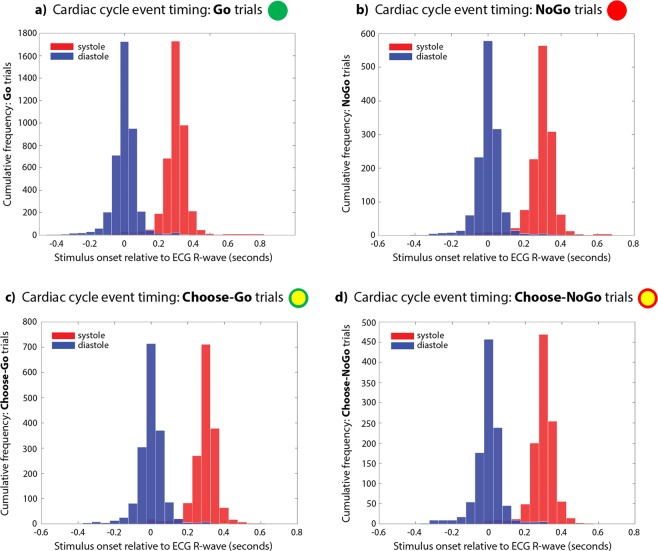
Precision of cue timing within the cardiac cycle, relative to the R-wave peak, in 50 ms time bins, for (**a**) Go, (**b**) NoGo, (**c**) Choose-Go, and (**d**) Choose-NoGo trials. Over 90% of trials were within 200 ms of the intended timing for diastole trials at 10 ms prior to the R-wave (blue), and for systole trials at 290 ms following R-wave (red). Dark blue indicates overlap of diastole and systole trial timings (minimal for all trial types).

To determine the achieved precision of trial event timing within the cardiac cycle, we used in-house Spike2 and Matlab scripts to extract the stimulus onsets from the Spike recording, and plot their relative position to the R-wave peak, in 50 ms time bins (Fig. 2). Dynamic monitoring of the inter-beat interval throughout the task ensured accurate synchronisation of the task cues with specified cardiac events, even when participants’ heart rates may vary over time, with attention, fatigue, and potentially, in response to stimuli and to action execution or inhibition^23–25^.

### Interoception heartbeat tracking task

Individual differences in interoceptive sensitivity were quantified using a heartbeat perception task. Participants’ heartbeats were monitored using a pulse oximeter attached to their left index finger (‘soft’ mount PureLight sensor; Nonin Medical Inc, MN, USA). Participants were instructed to “silently count the number of heartbeats you feel from the time you hear ‘start’ to when you hear ‘stop’”, on six trials of varying duration (25, 30, 35, 40, 45, 50 s), presented in a randomised order^41^. Following each trial, participants gave a confidence rating in their reported perceived number of heartbeats on a visual analogue scale, from ‘total guess (no heartbeat awareness)’ to ‘complete confidence (full perception of heartbeat’), scored from 0 (no heartbeat awareness) to 10 (full perception of heartbeat).

### Dimensions of interoception

We summarise the interoceptive indices calculated in this study and the dimensions of interoception to which they belong in Table 1.

**Table 1:** Interoceptive indices calculated in this study, the dimensions of interoception to which they belong, task employed, which accuracy equation is used to calculate the values, and whether the index represents a state or trait assessment of interoception.

Index	‘Standard’ accuracy	‘Alternative’ accuracy	Insight	Awareness	Confidence	Sensibility	Trait interoceptive prediction error (TIPE)
Dimension	Accuracy	Accuracy	Metacognition / State discrepancy	Metacognition		Sensibility	Trait discrepancy
Task	Heartbeat tracking	Heartbeat tracking	Heartbeat tracking & confidence	Heartbeat tracking & confidence	Confidence	Body Perception Questionnaire	Heartbeat tracking & Body Perception Questionnaire
Accuracy calculation method	‘Standard’ accuracy	‘Alternative’ accuracy	‘Standard’ accuracy	‘Alternative’ accuracy			‘Alternative’ accuracy
State or trait	State	State	State	State	State	Trait	Trait

### Interoceptive accuracy

Interoceptive accuracy, reflecting objective interoceptive performance, was calculated using an in-house Matlab script according to the trial-by-trial ratio of perceived to actual heartbeats, with two equations: (1 - (nbeats_real_ – nbeats_reported_) / nbeats_real_) (‘standard’ accuracy), and (1 – (nbeats_real_ – nbeats_reported_) / (nbeats_real_ + nbeats_reported_) / 2) (‘alternative’ accuracy)^26,27,42^. For each accuracy method, the ratios for the six trials were averaged to give a mean heartbeat tracking score. The ‘standard’ accuracy calculation is appropriate only in cases where participants do not estimate more than 2x the number of real heartbeats. When participants do substantially overestimate, the ‘alternative’ accuracy calculation is more appropriate^26,27^. One participant was a substantial over-estimator, according to this heuristic: we therefore report ‘standard’ accuracy scores for the remaining 40 participants, and ‘alternative’ accuracy scores for the full sample of 41 participants.

### Interoceptive awareness and insight

Interoceptive awareness and insight, reflecting metacognitive perception of one’s interoceptive accuracy performance, were calculated using an in-house Matlab script, according to two approaches: a discrepancy index between the participant’s accuracy and confidence scores (using ‘standard’ accuracy scores), and the Pearson correlation between interoceptive accuracy and confidence rating on each trial (using ‘alternative’ accuracy scores)^26,27^. To calculate the discrepancy index (insight), confidence ratings were converted from a 0 to 10 scale to a percentage (confidence rating / 10), then an absolute difference score calculated for each trial (‘standard’ accuracy – confidence), which was averaged across the six trials. Because the ‘alternative’ accuracy calculation method can generate negative values, it was possible to apply the discrepancy index to ‘standard’ accuracy scores only from the 40 participants who did not substantially overestimate their perceived heartbeats. The within-participant correlation between accuracy and confidence scores (awareness) was calculated using ‘alternative’ accuracy scores in all 41 participants.

We describe the discrepancy index as ‘interoceptive insight’ and the correlation measure as ‘interoceptive awareness’, following the original terminology of the authors who introduced these methods^26,27^. An insight discrepancy score of 0 indicates high interoceptive metacognition, with optimum alignment of accuracy and confidence (accuracy exactly matches confidence rating). The further the discrepancy score from 0, the poorer the interoceptive metacognition, with negative discrepancy scores indicating low performance but high confidence, and positive discrepancy scores indicating high performance but low confidence. In contrast, low (negative) awareness correlation scores indicate poor interoceptive metacognition, while high (positive) awareness correlation scores indicate better interoceptive metacognition.

In addition to the metacognitive measures of interoceptive awareness and insight, we also calculated the mean confidence rating on the heartbeat tracking task, to provide a general indication of confidence, separate to interoceptive processes.

### Interoceptive sensibility

Participants completed the awareness section of the Body Perception Questionnaire^43^. The BPQ was used to calculate (trait) interoceptive sensibility, reflecting self-reported subjective sensitivity to bodily sensations, according to the mean score of the response to the 45 items (each scored from 1 to 5).

### Trait interoceptive prediction error (TIPE)

Heartbeat tracking accuracy (‘alternative’ scores) and BPQ scores were converted to standardised z-scores (SPSS), and TIPE calculated as the discrepancy between z-scored accuracy and z-scored sensibility (z-sensibility – z-accuracy). Positive TIPE values reflect a tendency for individuals to *over-estimate* interoceptive ability, while negative values reflect a tendency to *under-estimate*^28^.

### Heart rate variability

To calculate heart rate variability (HRV), ECG recording continued for 2.5 minutes following the intentional inhibition task, during which participants were instructed to rest quietly with eyes open. Each participant’s recording was visually inspected to confirm no movement artifacts, then thresholded to isolate each R-wave peak, and the inter-beat intervals calculated using in-house Spike2 and Matlab scripts. The inter-beat intervals were entered to the HRVAS toolbox^44^ in Matlab. Heart rate variability was indexed as the root mean square of the successive differences (RMSSD). The HRVAS output also provided heart rate in beats per minute (bpm), which was entered to multiple regressions as a confounding factor.

### Trait impulsivity

Participants completed three questionnaires indexing facets of trait impulsivity: the Barratt Impulsivity Scale (BIS)^45^, giving a total and three attention, motor, and planning subscale scores; the Conners Adult ADHD Rating Scale – Short Scale (CAARS-SS)^46^, giving an index of ADHD symptomology; and the UPPS-P^47^, giving five subscale scores on negative urgency, lack of premeditation, lack of perseverance, sensation seeking, and positive urgency.

### Statistical analyses

Data were analysed in JASP (https://jasp-stats.org/), using both frequentist tests and Bayesian equivalents, extending insights to guide interpretation of significance (p values) according to how likely the alternative hypothesis is versus the null (BF_10_). For t-tests, in addition to the statistic and significance value, we give the 95% confidence intervals of the mean difference. For the Bayesian analyses, in the absence of pilot data on relationships amongst intentional inhibition, cardiac cycle, and dimensions of interoception, we used the default JASP priors, which assume a medium effect size on a Cauchy distribution of 0.707 for t-tests, beta binomials of 1 for multiple regressions, and stretched beta prior widths of 1 for correlations. The.jasp file, containing the data, analysis options, and output, is available at https://osf.io/3xezy. This also contains graphical robustness checks examining evidence for H_1_ and H_0_ under different prior widths, for t-tests. We interpret Bayes Factors (BF_10_) according to the heuristic of 1–3 indicating anecdotal evidence in favour of H_1_, 3–10 moderate evidence, and >10 strong evidence^48^.

Firstly, to compare behaviour on the intentional inhibition task between systole and diastole trials, we used four 2-tailed repeated measures t-tests, for (1) Go reaction times, (2) % NoGo commission errors, (3) % of Choose trials on which participants chose to go (% Choose-Go), and (4) reaction times on Choose-Go trials. We report 95% confidence intervals of the mean difference. While participants’ intentional inhibition rates (% Choose-Go), and reactive inhibition in the form of the NoGo trials, were the primary measures of interest, we include reaction time analyses to provide complementary analyses of different facets of motor behaviour. The %NoGo commission error data were not normally distributed, and so for this comparison we report the results of a non-parametric Wilcoxon-test.

Secondly, to investigate how individual differences in interoception relate to motor behaviour, we generated a correlation matrix of 2-tailed Pearson correlations between all seven interoceptive indices and four motor behaviour measures (Go reaction times, % NoGo errors, % Choose-Go, Choose-Go reaction times).

Thirdly, to investigate the influence of HRV on motor behaviour, we used four linear regressions, with (1) Go reaction time, (2) % NoGo commission errors, (3) % Choose-Go, and (4) Choose-Go reaction time as the dependent variable, and RMSSD and beats per minute as the independent variables, using the ‘Enter’ method (which does not assume a stronger contribution of any one of the independent variables in the prediction of the dependent variable). Bayesian regressions compared a full inclusion model to the null model.

Fourthly, to explore how individual differences in trait impulsivity relate to motor behaviour, we generated a correlation matrix of 2-tailed Pearson correlations between the four behavioural measures on the intentional inhibition task (Go reaction times, % NoGo errors, % Choose-Go, Choose-Go reaction times), and the impulsivity questionnaire scores (BIS: total, and the attention, motor, and planning subscales; CAARS-SS ADHD index; and the five subscales of the UPPS-P).

Finally, to investigate how individual differences in interoception relate to trait impulsivity, we generated a correlation matrix of 2-tailed Pearson correlations between the seven interoceptive indices and a subset of the impulsivity questionnaire scores (BIS: total, attention, motor, planning; CAARS-SS ADHD index).

## Results

Descriptive statistics (mean, standard deviation, minimum and maximum) for all experimental measures are given in Table 2.

**Table 2:** Demographic details of participants, and measures of performance on the cardiac intentional inhibition task, dimensions of interoception, trait impulsivity, and heart rate variability.

Category	Variable	Mean	Standard deviation	Minimum	Maximum
Age		22	2	18	28
Cardiac intentional inhibition task	Go reaction time: systole (ms)	483	59	352	623
	Go reaction time: diastole (ms)	481	56	360	607
	Go reaction time: mean (ms)	482	57	356	615
	% Go omissions: systole	2	2	0	9
	% Go omissions: diastole	2	2	0	13
	% Go omissions: mean	1	2	0	5
	% NoGo errors: systole	4	5	0	18
	% NoGo errors: diastole	4	6	0	27
	% NoGo errors: mean	4	5	0	18
	NoGo reaction time: systole (ms)	408	72	299	546
	NoGo reaction time: diastole (ms)	397	62	289	522
	NoGo reaction time: mean (ms)	405	61	294	538
	% Choose-Go: systole	60	13	37	87
	% Choose-Go: diastole	61	11	40	84
	% Choose-Go: mean	60	11	42	84
	Choose-Go reaction time: systole (ms)	524	86	357	712
	Choose-Go reaction time: diastole (ms)	522	81	360	686
	Choose-Go reaction time: mean (ms)	523	82	359	689
Interoception	Heartbeat tracking accuracy (‘Standard’)	0.71	0.18	0.24	0.97
	Heartbeat tracking accuracy (‘Alternative’)	0.62	0.29	−0.23	0.97
	Heartbeat tracking insight (accuracy-confidence discrepancy)	0.24	0.21	−0.29	0.69
	Heartbeat tracking awareness (accuracy-confidence correlation)	0.26	0.48	−0.88	0.98
	Confidence	4.71	1.92	0.85	7.87
	Sensibility (BPQ)	2.5	0.64	1.49	3.89
	Trait interoceptive prediction error (TIPE)	<0.01	1.39	−2.81	3.81
BIS	Attention	18	4	11	26
	Motor	24	4	16	32
	Planning	24	5	14	36
	Total	66	11	49	86
CAARS-SS	ADHD index	13	4	4	21
UPPS-P	Negative urgency	29	7	16	42
	Premeditation	23	4	14	30
	Perseverance	20	3	14	30
	Sensation seeking	38	7	21	51
	Positive urgency	29	9	16	49
Heart rate variability	RMSSD	54	29	15	175
	Beats per minute (bpm)	75	10	53	95

### Cardiac intentional inhibition task

**Figure 3:**
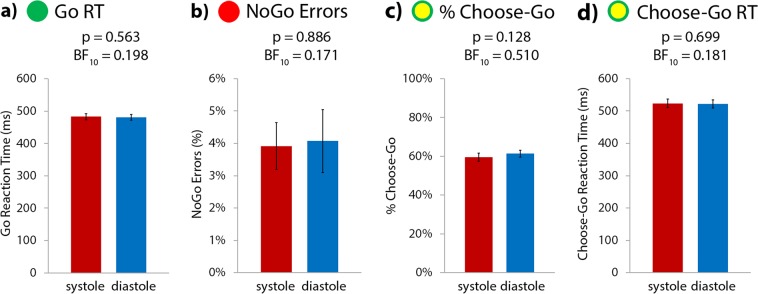
No significant difference between systole and diastole for (**a**) reaction time on Go trials, (**b**) % errors on NoGo trials, (**c**) frequency of choosing to go on Choose trials (% Choose-Go), or (**d**) reaction time on Choose-Go trials. Error bars represent standard error of the mean (SEM).

There was no significant effect of cardiac timing on motor behaviour in the intentional inhibition task, on any of the four task measures (Fig. 3).

Go reaction time was not significantly different at systole (483 ms) to diastole (481 ms) (t(40) = 0.583, p = 0.563, mean difference = 2 ms [−4, 7], with BF_10_ = 0.198, suggesting moderate evidence for H_0_).

Participants did not make commission errors any more frequently on NoGo trials at systole (4%) than diastole (4%) (Z = 224.500, p = 0.886, mean difference = <1% [−3, 3], with BF_10_ = 0.171, suggesting moderate evidence for H_0_). However, we interpret this comparison with caution, given that on the whole, participants made very few NoGo errors, with a mean NoGo error rate of 4% – equating to an average of only 3 NoGo errors per participant throughout the experiment – while 10 participants did not make a single commission error at all. This means that paucity of NoGo error trials, leading to floor effects, could obscure any potential cardiac effect.

On the critical trial type of interest, Choose trials - when participants selected whether to act or to inhibit their button press - how frequently they chose to go (% Choose-Go) was not significantly different at systole (60%) to diastole (61%) (t(40) = −1.553, p = 0.128, mean difference = 2% [−4, 1], with BF_10_ = 0.510, suggesting anecdotal evidence for H_0_).

On Choose trials where participants elected to go, reaction times were not significantly different when the Choose cue had been presented at systole (524 ms) to diastole (522 ms) (t(40) = 0.390, p = 0.699, mean difference = 2 ms [−7, 11], with BF_10_ = 0.181, suggesting moderate evidence for H_0_).

### Dimensions of interoception

**Figure 4:**
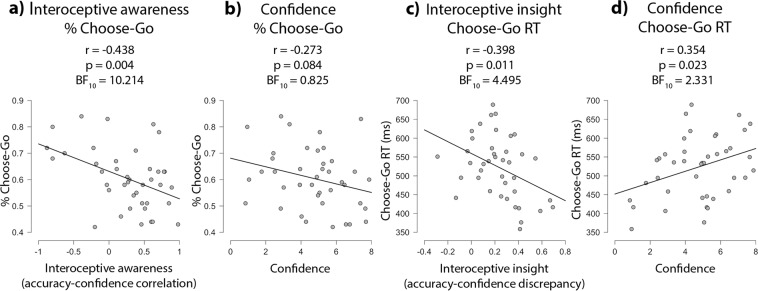
Correlations of interoceptive awareness (accuracy-confidence correlation), interoceptive insight (accuracy-confidence discrepancy), and confidence with how frequently participants chose to go on Choose trials (% Choose-Go) and their reaction times. Low (negative) accuracy-confidence correlation scores indicate poor interoceptive awareness, while high (positive) accuracy-confidence correlation scores indicate better interoceptive awareness. An insight score of 0 indicates high interoceptive metacognition, with optimum alignment of accuracy and confidence. The further the discrepancy score from 0, the poorer the interoceptive metacognition, with negative discrepancy scores indicating low performance but high confidence, and positive discrepancy scores indicating high performance but low confidence.

A correlation matrix investigated how individual differences in interoception relate to motor behaviour (Table 3). There were no significant associations between interoception and Go reaction times or % NoGo errors. However, interoceptive awareness correlated negatively with % Choose-Go (r = −0.438, p = 0.004, BF_10_ = 10.214), such that the lower the interoceptive awareness, the more frequently participants decided to act on Choose trials (Fig. 4a). A weak negative correlation between heartbeat tracking confidence ratings and % Choose-Go (r = −0.273, p = 0.084, BF_10_ = 0.825) that did not reach statistical significance suggested this may be partly explained by participants who have lower confidence choosing to act more frequently (Fig. 4b). Interestingly, interoceptive insight did not relate to % Choose-Go, but was associated with reaction times on Choose-Go trials (r = −0.398, p = 0.011, BF_10_ = 4.495), such that the poorer the interoceptive insight (higher discrepancy scores), the faster the reaction time (Fig. 4c). A significant correlation between confidence ratings and Choose-Go reaction times (r = 0.354, p = 0.023, BF_10_ = 2.331) suggests the association with interoceptive insight may be partly explained by participants who have lower confidence responding more quickly (Fig. 4d).

**Table 3:** Correlations (2-tailed) between performance on the intentional inhibition task, and interoceptive indices. Significant (p < 0.05) correlations highlighted in bold.

	Go RT	% NoGo error	% Choose-Go	Choose-Go RT	‘Standard’ accuracy	‘Alternative’ accuracy	Insight	Awareness	Confidence	Sensibility	Trait interoceptive prediction error (TIPE)
Go RT											
% NoGo error	*r* −0.394 ***p*** **0.011** BF_10_ 4.516										
% Choose-Go	*r* −0.213 *p* 0.182 BF_10_ 0.461	*r* 0.170 *p* 0.287 BF_10_ 0.336									
Choose-Go RT	*r* 0.748 ***p*** < **0.001** BF_10_ 804247.390	*r* −0.330 ***p*** **0.035** BF_10_ 1.660	*r* −0.353 ***p*** **0.024** BF_10_ 2.308								
‘Standard’ accuracy	*r* −0.101 *p* 0.534 BF_10_ 0.237	*r* 0.028 *p* 0.866 BF_10_ 0.200	*r* −0.081 *p* 0.620 BF_10_ 0.222	*r* −0.084 *p* 0.604 BF_10_ 0.224							
‘Alternative’ accuracy	*r* −0.095 *p* 0.553 BF_10_ 0.231	*r* 0.039 *p* 0.807 BF_10_ 0.200	*r* 0–0.107 *p* 0.505 BF_10_ 0.241	*r* −0.093 *p* 0.563 BF_10_ 0.229	*r* 0.991 ***p*** < **0.001** BF_10_ ∞						
Insight	*r* −0.250 *p* 0.119 BF_10_ 0.635	*r* 0.087 *p* 0.593 BF_10_ 0.226	*r* 0.187 *p* 0.248 BF_10_ 0.375	*r* −0.398 ***p*** **0.011** BF_10_ 4.495	*r* 0.523 ***p*** < **0.001** BF_10_ 63.674	*r* 0.539 ***p*** < **0.001** BF_10_ 97.196					
Awareness	*r* −0.084 *p* 0.602 BF_10_ 0.222	*r* 0.211 *p* 0.186 BF_10_ 0.452	*r* −0.438 ***p*** **0.004** BF_10_ 10.214	*r* −0.011 *p* 0.948 BF_10_ 0.195	*r* 0.329 ***p*** **0.038** BF_10_ 1.574	*r* 0.326 ***p*** **0.037** BF_10_ 1.579	*r* −0.013 *p* 0.934 BF_10_ 0.198				
Confidence	*r* 0.172 *p* 0.281 BF_10_ 0.341	*r* −0.069 *p* 0.667 BF_10_ 0.213	*r* −0.273 *p* 0.084 BF_10_ 0.825	*r* 0.354 ***p*** **0.023** BF_10_ 2.331	*r* 0.376 ***p*** **0.017** BF_10_ 3.120	*r* 0.343 ***p*** **0.028** BF_10_ 1.995	*r* −0.593 ***p*** < **0.001** BF_10_ 500.456	*r* 0.326 ***p*** **0.037** BF_10_ 1.576			
Sensibility	*r* 0.195 *p* 0.222 BF_10_ 0.400	*r* −0.027 *p* 0.866 BF_10_ 0.197	*r* −0.009 *p* 0.957 BF_10_ 0.195	*r* 0.079 *p* 0.625 BF_10_ 0.218	*r* 0.046 *p* 0.780 BF_10_ 0.205	*r* 0.035 *p* 0.830 BF_10_ 0.199	*r* −0.027 *p* 0.866 BF_10_ 0.200	*r* 0.123 *p* 0.445 BF_10_ 0.258	*r* 0.070 *p* 0.664 BF_10_ 0.213		
Trait interoceptive prediction error (TIPE)	*r* 0.209 *p* 0.190 BF_10_ 0.446	*r* −0.048 *p* 0.768 BF_10_ 0.203	*r* 0.071 *p* 0.661 BF_10_ 0.214	*r* 0.124 *p* 0.441 BF_10_ 0.259	*r* −0.671 ***p*** < **0.001** BF_10_ 10026.857	*r* −0.694 ***p*** < **0.001** BF_10_ 39767.605	*r* −0.403 ***p*** **0.010** BF_10_ 4.849	*r* −0.146 *p* 0.362 BF_10_ 0.291	*r* −0.196 *p* 0.220 BF_10_ 0.403	*r* 0.695 ***p*** < **0.001** BF_10_ 41427.547

### Heart rate variability

Four multiple regressions tested for an influence of HRV (RMSSD) and heart rate (bpm) on motor behaviour. There was no significant relation between HRV, bpm, and Go reaction times (F(2,38) = 1.153, p = 0.327, with BF_10_ = 0.311), nor % NoGo errors (F(2,38) = 0.401, p = 0.672, with BF_10_ = 0.179), nor % Choose-Go (F(2,38) = 1.439, p = 0.250, with BF_10_ = 0.383), or Choose-Go reaction times (F(2,38) = 0.993, p = 0.380, with BF_10_ = 0.277).

### Trait impulsivity

We generated two correlation matrices of 2-tailed Pearson correlations between 1) the four behavioural measures on the intentional inhibition task, and impulsivity questionnaire scores (Table 4), and 2) the seven interoceptive indices and a subset of the impulsivity questionnaire scores (Table 5).

**Table 4:** Correlations (2-tailed) between performance on the intentional inhibition task, and impulsivity questionnaire scores. Significant (p < 0.05) correlations highlighted in bold.

	Go RT	% NoGo error	% Choose-Go	Choose-Go RT	BIS attention	BIS motor	BIS planning	BIS total	CAARS-SS ADHD index	Negative urgency	Pre-meditation	Per-severance	Sensation seeking	Positive urgency
Go RT														
% NoGo error	*r* −0.394 ***p*** **0.011** BF_10_ 4.516													
% Choose-Go	*r* −0.213 *p* 0.182 BF_10_ 0.461	*r* 0.170 *p* 0.287 BF_10_ 0.336												
Choose-Go RT	*r* 0.748 ***p*** < **0.001** BF_10_ 804247.390	*r* −0.330 ***p*** **0.035** BF_10_ 1.660	*r* −0.353 ***p*** **0.024** BF_10_ 2.308											
BIS attention	*r* −0.294 *p* 0.062 BF_10_ 1.043	*r* 0.131 *p* 0.416 BF_10_ 0.268	*r* 0.104 *p* 0.516 BF_10_ 0.238	*r* −0.033 *p* 0.838 BF_10_ 0.199										
BIS motor	*r* −0.303 *p* 0.054 BF_10_ 1.171	*r* 0.122 *p* 0.446 BF_10_ 0.257	*r* 0.012 *p* 0.939 BF_10_ 0.195	*r* 0.022 *p* 0.892 BF_10_ 0.196	*r* 0.545 ***p*** < **0.001** BF_10_ 136.533									
BIS planning	*r* −0.333 ***p*** **0.034** BF_10_ 1.720	*r* 0.161 *p* 0.313 BF_10_ 0.318	*r* 0.051 *p* 0.750 BF_10_ 0.204	*r* −0.154 *p* 0.336 BF_10_ 0.304	*r* 0.343 ***p*** **0.028** BF_10_ 2.003	*r* 0.544 ***p*** < **0.001** BF_10_ 133.432								
BIS total	*r* −0.385 ***p*** **0.013** BF_10_ 3.857	*r* 0.173 *p* 0.280 BF_10_ 0.342	*r* 0.065 *p* 0.687 BF_10_ 0.210	*r* −0.077 *p* 0.634 BF_10_ 0.217	*r* 0.728 ***p*** < **0.001** BF_10_ 238571.500	*r* 0.861 ***p*** < **0.001** BF_10_ 1.513e + 10	*r* 0.824 ***p*** < **0.001** BF_10_ 2.940e + 8							
CAARS-SS ADHD index	*r* −0.425 ***p*** **0.006** BF_10_ 7.914	*r* 0.213 *p* 0.180 BF_10_ 0.463	*r* 0.286 *p* 0.070 BF_10_ 0.950	*r* −0.155 *p* 0.333 BF_10_ 0.306	*r* 0.647 ***p*** < **0.001** BF_10_ 4716.556	*r* 0.527 ***p*** < **0.001** BF_10_ 81.839	*r* 0.587 ***p*** < **0.001** BF_10_ 492.847	*r* 0.719 ***p*** < **0.001** BF_10_ 148176.743						
Negative urgency	*r* −0.078 *p* 0.626 BF_10_ 0.218	*r* 0.160 *p* 0.317 BF_10_ 0.316	*r* 0.362 ***p*** **0.020** BF_10_ 2.645	*r* −0.009 *p* 0.953 BF_10_ 0.195	*r* 0.490 ***p*** **0.001** BF_10_ 32.307	*r* 0.279 *p* 0.077 BF_10_ 0.878	*r* 0.320 ***p*** **0.041** BF_10_ 1.460	*r* 0.435 ***p*** **0.004** BF_10_ 9.679	*r* 0.600 ***p*** < **0.001** BF_10_ 786.692					
Premeditation	*r* 0.009 *p* 0.955 BF_10_ 0.195	*r* −0.062 *p* 0.702 BF_10_ 0.209	*r* −0.182 *p* 0.256 BF_10_ 0.363	*r* −0.036 *p* 0.823 BF_10_ 0.199	*r* 0.309 ***p*** **0.049** BF_10_ 1.264	*r* 0.490 ***p*** **0.001** BF_10_ 32.363	*r* 0.444 ***p*** **0.004** BF_10_ 11.501	*r* 0.522 ***p*** < **0.001** BF_10_ 71.666	*r* 0.341 ***p*** **0.029** BF_10_ 1.946	*r* 0.173 *p* 0.281 BF_10_ 0.341				
Perseverance	*r* −0.292 *p* 0.064 BF_10_ 1.022	*r* −0.141 *p* 0.381 BF_10_ 0.282	*r* −0.125 *p* 0.438 BF_10_ 0.260	*r* −0.013 *p* 0.935 BF_10_ 0.195	*r* 0.277 *p* 0.080 BF_10_ 0.857	*r* 0.278 *p* 0.079 BF_10_ 0.868	*r* 0.319 ***p*** **0.042** BF_10_ 1.426	*r* 0.362 ***p*** **0.020** BF_10_ 2.663	*r* 0.288 *p* 0.068 BF_10_ 0.970	*r* 0.082 *p* 0.609 BF_10_ 0.221	*r* 0.277 *p* 0.080 BF_10_ 0.857			
Sensation seeking	*r* −0.054 *p* 0.736 BF_10_ 0.206	*r* 0.063 *p* 0.694 BF_10_ 0.210	*r* −0.084 *p* 0.603 BF_10_ 0.222	*r* 0.073 *p* 0.650 BF_10_ 0.215	*r* 0.445 ***p*** **0.004** BF_10_ 11.901	*r* 0.555 ***p*** < **0.001** BF_10_ 181.521	*r* 0.258 *p* 0.103 BF_10_ 0.702	*r* 0.505 ***p*** < **0.001** BF_10_ 45.800	*r* 0.314 ***p*** **0.045** BF_10_ 1.343	*r* 0.391 ***p*** **0.011** BF_10_ 4.262	*r* 0.478 ***p*** **0.002** BF_10_ 24.001	*r* 0.034 *p* 0.835 BF_10_ 0.199		
Positive urgency	*r* −0.341 ***p*** **0.029** BF_10_ 1.951	*r* 0.439 ***p*** **0.004** BF_10_ 10.450	*r* 0.224 *p* 0.159 BF_10_ 0.507	*r* −0.104 *p* 0.519 BF_10_ 0.238	*r* 0.670 ***p*** < **0.001** BF_10_ 12697.285	*r* 0.604 ***p*** < **0.001** BF_10_ 890.075	*r* 0.457 ***p*** **0.003** BF_10_ 15.039	*r* 0.696 ***p*** < **0.001** BF_10_ 43698.378	*r* 0.678 ***p*** < **0.001** BF_10_ 18082.328	*r* 0.674 ***p*** < **0.001** BF_10_ 14984.850	*r* 0.214 *p* 0.179 BF_10_ 0.466	*r* 0.190 *p* 0.235 BF_10_ 0.384	*r* 0.580 ***p*** < **0.001** BF_10_ 394.4

**Table 5:** Correlations (2-tailed) between interoceptive indices, and impulsivity questionnaire scores. Significant (p < 0.05) correlations highlighted in bold.

	‘Standard’ accuracy	‘Alternative’ accuracy	Insight	Awareness	Confidence	Sensibility	Trait interoceptive prediction error (TIPE)	BIS attention	BIS motor	BIS planning	BIS total	CAARS-SS ADHD index
‘Standard’ accuracy												
‘Alternative’ accuracy	*r* 0.991 ***p*** < **0.001** BF_10_ ∞											
Insight	*r* 0.523 ***p*** < **0.001** BF_10_ 63.674	*r* 0.539 ***p*** < **0.001** BF_10_ 97.196										
Awareness	*r* 0.329 ***p*** **0.038** BF_10_ 1.574	*r* 0.326 ***p*** **0.037** BF_10_ 1.579	*r* −0.013 *p* 0.934 BF_10_ 0.198									
Confidence	*r* 0.376 ***p*** **0.017** BF_10_ 3.120	*r* 0.343 ***p*** **0.028** BF_10_ 1.995	*r* −0.593 ***p*** < **0.001** BF_10_ 500.456	*r* 0.326 ***p*** **0.037** BF_10_ 1.576								
Sensibility	*r* 0.046 *p* 0.780 BF_10_ 0.205	*r* 0.035 *p* 0.830 BF_10_ 0.199	*r* −0.027 *p* 0.866 BF_10_ 0.200	*r* 0.123 *p* 0.445 BF_10_ 0.258	*r* 0.070 *p* 0.664 BF_10_ 0.213							
Trait interoceptive prediction error (TIPE)	*r* −0.671 ***p*** < **0.001** BF_10_ 10026.857	*r* −0.694 ***p*** < **0.001** BF_10_ 39767.605	*r* −0.403 ***p*** **0.010** BF_10_ 4.849	*r* −0.146 *p* 0.362 BF_10_ 0.291	*r* −0.196 *p* 0.220 BF_10_ 0.403	*r* 0.695 ***p*** < **0.001** BF_10_ 41427.547						
BIS attention	*r* 0.216 *p* 0.181 BF_10_ 0.468	*r* 0.196 *p* 0.220 BF_10_ 0.402	*r* 0.067 *p* 0.681 BF_10_ 0.214	*r* 0.361 ***p*** **0.021** BF_10_ 2.598	*r* 0.132 *p* 0.411 BF_10_ 0.270	*r* 0.134 *p* 0.402 BF_10_ 0.273	*r* −0.044 *p* 0.784 BF_10_ 0.202					
BIS motor	*r* 0.296 *p* 0.064 BF_10_ 1.037	*r* 0.290 *p* 0.065 BF_10_ 1.002	*r* −0.041 *p* 0.804 BF_10_ 0.203	*r* 0.135 *p* 0.401 BF_10_ 0.274	*r* 0.324 ***p*** **0.039** BF_10_ 1.524	*r* 0.125 *p* 0.437 BF_10_ 0.261	*r* −0.119 *p* 0.460 BF_10_ 0.253	*r* 0.545 ***p*** < **0.001** BF_10_ 136.533				
BIS planning	*r* 0.004 *p* 0.978 BF_10_ 0.197	*r* 0.006 *p* 0.969 BF_10_ 0.195	*r* 0.076 *p* 0.642 BF_10_ 0.219	*r* −0.064 *p* 0.690 BF_10_ 0.210	*r* −0.077 *p* 0.632 BF_10_ 0.217	*r* −0.142 *p* 0.375 BF_10_ 0.285	*r* −0.107 *p* 0.506 BF_10_ 0.241	*r* 0.343 ***p*** **0.028** BF_10_ 2.003	*r* 0.544 ***p*** < **0.001** BF_10_ 133.432			
BIS total	*r* 0.197 *p* 0.224 BF_10_ 0.402	*r* 0.189 *p* 0.236 BF_10_ 0.383	*r* 0.042 *p* 0.796 BF_10_ 0.203	*r* 0.146 *p* 0.362 BF_10_ 0.290	*r* 0.414 *p* 0.378 BF_10_ 0.283	*r* 0.028 *p* 0.861 BF_10_ 0.197	*r* −0.116 *p* 0.471 BF_10_ 0.250	*r* 0.728 ***p*** < **0.001** BF_10_ 238571.500	*r* 0.861 ***p*** < **0.001** BF_10_ 1.513e + 10	*r* 0.824 ***p*** < **0.001** BF_10_ 2.940e + 8		
CAARS-SS ADHD index	*r* 0.032 *p* 0.845 BF_10_ 0.201	*r* 0.045 *p* 0.779 BF_10_ 0.202	*r* 0.111 *p* 0.495 BF_10_ 0.247	*r* −0.034 *p* 0.834 BF_10_ 0.199	*r* −0.084 *p* 0.600 BF_10_ 0.222	*r* −0.023 *p* 0.886 BF_10_ 0.196	*r* −0.023 *p* 0.886 BF_10_ 0.204	*r* 0.647 ***p*** < **0.001** BF_10_ 4716.556	*r* 0.527 ***p*** < **0.001** BF_10_ 81.839	*r* 0.587 ***p*** < **0.001** BF_10_ 492.847	*r* 0.719 ***p*** < **0.001** BF_10_ 148176.743	

Go reaction times correlated negatively with BIS planning (r = −0.333, p = 0.034, BF_10_ = 1.720), BIS total (r = −0.385, p = 0.013, BF_10_ = 3.857), CAARS-SS ADHD index (r = −0.425, p = 0.006, BF_10_ = 7.914), and UPPS-P positive urgency (r = −0.341, p = 0.029, BF_10_ = 1.951), such that the greater the impulsivity questionnaire score, the shorter the Go reaction time. NoGo errors correlated positively with UPPS-P positive urgency (r = 0.439, p = 0.004, BF_10_ = 10.450), such that the greater a participant’s positive urgency (reflecting a tendency to act impulsively while experiencing strong positive emotions), the more frequently they made NoGo errors. Finally, % Choose-Go correlated positively with UPPS-P negative urgency (r = 0.362, p = 0.020, BF_10_ = 2.645), such that the greater a participant’s negative urgency (reflecting a tendency to act impulsively while experiencing strong negative emotions), the more often they chose to act on Choose trials.

Interoceptive awareness correlated positively with BIS attention (r = 0.361, p = 0.021, BF_10_ = 2.598), and confidence ratings correlated positively with BIS motor (r = 0.324, p = 0.039, BF_10_ = 1.524), such that the greater a participant’s attentional impulsivity, the higher their interoceptive awareness, and the greater their motor impulsivity, the higher their confidence on the heartbeat tracking task.

## Discussion

Physiological signals from the body influence emotion, cognition, and action^13,15,49^. This includes heartbeats, which can cue more efficient response inhibition^18^. We tested whether these effects extend to intentional inhibition, in which participants choose whether to act, or to withhold movements, with Choose cues presented at either cardiac systole or diastole. In fact, we found no conclusive evidence that choice cues presented at systole led to more frequent choices to withhold actions than choice cues presented at diastole. However, individual differences in interoceptive ability – specifically, interoceptive awareness and insight – were related to participants’ intentional inhibition rates and speed of decision. Participants with better interoceptive awareness and insight tended to choose to withhold actions and respond more slowly, while participants with poorer interoceptive awareness and insight tended to choose to execute actions and respond faster, suggesting lower (metacognitive) insight into bodily perception is connected to feelings of urges to move the body.

We also investigated whether autonomic and neurocognitive endophenotype traits, as measured by heart rate variability and self-report impulsivity questionnaires, were related to motor behaviour and interoceptive indices. We found little compelling evidence to that effect. Our primary observations were that faster Go reaction times were associated with greater impulsivity across a range of scales; NoGo error and Choose-Go rates with feelings of urgency on the UPPS-P; and higher BIS subscale trait impulsivity with greater interoceptive awareness and state sensibility (confidence), albeit with Bayes Factors that were often anecdotal.

### Cardiac cues to inhibitory processes

When the heart contracts, baroreceptors in blood vessel walls signal the strength and timing of heartbeats to the central nervous system^10^. During states of high cardiovascular arousal, when heightened attention to salient stimuli and reprioritisation of action plans are important, increased baroreceptor signalling to brainstem and thalamic relay nuclei, then insular cortex, update the central representation of bodily physiology^11,15^. Fast homeostatic readjustments of behaviour are often required in scenarios of heightened autonomic arousal to adjust sensory perception and trigger aversive or avoidant reactions. On this basis, one can predict that cardiac signals would enhance inhibitory control. Indeed a broad invocation of inhibitory processes across behavioural domains at cardiac systole has been asserted historically^22^.

In the domain of reactive response inhibition, we previously found that stopping efficiency, as measured by stop signal reaction times on the stop signal task, was enhanced if the stop cues were presented at cardiac systole^18^. Here, we extend this observation from reactive motor inhibition to examine the volitional withholding of actions, i.e. ‘intentional inhibition’^5^. Interestingly, this form of motor inhibition was unaffected by cardiac cycle. Participants were equally likely to choose to act, or to withhold actions, when Choose cues were presented at systole and diastole. This carries a number of interesting implications.

Firstly, it may be that momentary state signals representing cardiovascular arousal are important cues to invoke the fast, rapid, reactive motor inhibition required during the stop signal task – and during threatening, or even dangerous, scenarios in everyday life, while less important for internally generated choices to act or to withhold motor plans. These action processes may be driven by sustained goal-oriented cognitive sets rather than momentary fluctuations in arousal state. Alternatively, it is possible that physiological state cues do still cue intentional inhibition, but the artificiality of tasks that require participants to make volitional choices ‘on cue’ in an experimental context obscures our sensitivity to measure truly voluntary action. We used a modified Go/NoGo task, incorporating Choose trials on which participants chose whether to ‘Go’ or ‘NoGo’. This paradigm was applied previously to measure intentional inhibition^8,14^, but arguably has reduced ecological validity in relation to the intentional inhibition processes that occur in everyday life. The ‘marble’ task^50^, and pain avoidance task^7^, may better reflect such everyday intentional inhibition processes. However, these are challenging to implement in a ‘cardiac cycle study’ since trial lengths are typically longer than a typical cardiac cycle. Finally, there are substantial individual differences in feelings of urges to move, which may relate less to transient signalling of autonomic processes, and more to longer-lasting trait level phenotypes.

However, we note that the Bayes Factor comparing % Choose-Go between systole and diastole (0.510) suggested only anecdotal evidence in favour of the null hypothesis, and a larger sample size would likely enable us to more firmly conclude in favour of no impact of cardiac cycle on intentional inhibition.

By employing a modified Go/NoGo task, it was possible to simultaneously investigate the impact of cardiac cycle on reactive inhibition within the same participants. This provides a complementary investigation to our previous study employing the stop signal task^18^, since NoGo trials require participants to withhold a prepotent response, while stop signal trials require participants to cancel an action that has already been initiated. In contrast to our previous finding that reactive inhibition on the stop signal task is improved during systole, here we found no evidence for a similar effect on NoGo inhibition. However, we interpret this finding with caution since, here, the paucity of NoGo error trials likely led to floor effects, obscuring our ability to detect any potential cardiac influence. A more traditional NoGo task, i.e. not including the additional Choose trials, may better invoke the prepotent tendency to act and hence elicit a higher NoGo error rate to better study this form of reactive inhibition. Nevertheless, the number of NoGo error trials will inevitably remain small, and vary between participants. Thus, the stop signal task is better suited to investigations of reactive inhibition where minimum trial numbers are necessary to compare between cardiac states^18^.

### Individual differences in interoceptive awareness and insight

Although we found no conclusive evidence for an effect of cardiac cycle, we did observe some notable individual participant differences in intentional inhibition rates – with some choosing to move as infrequently as 40% on choice trials, and others as frequently as 80% – which correlated with interoceptive awareness. In addition, Choose-Go reaction times correlated with interoceptive insight (accuracy-confidence discrepancy), such that participants with poorer insight tended to move faster.

Interoceptive awareness and insight reflect the metacognitive perception of one’s interoceptive accuracy performance, according to confidence-accuracy correspondence. This suggests that while dynamic interoceptive cues (such as individual heartbeats) may not influence intentional motor decisions, higher-order (state-based) perception of the body forms one factor associated with frequency of volitional decisions to move or to inhibit.

Interoceptive awareness reflects ‘the ability of the individual to know when they are making good or bad interoceptive decisions’^26^, and may contribute to control of impulsive decision-making. A lack of insight into the accuracy of one’s ‘somatic markers’ can arise from prefrontal dysfunction (as opposed to damage to the insula, which impairs instead the representation and detection of such internal bodily changes). This can lead to increased risky decisions for short-term over long-term gain^51^. Our finding raises the possibility that ability to reflect on interoceptive sensitivity also embeds more elemental aspects of motor control by reducing feelings of urge to move. Thus, those who are better able to explain and accommodate changes in internal state through higher awareness are less likely to choose to act.

This process may operate via accumulation-of-evidence processes that underpin motor control and the release or inhibition of actions^52^. Voluntary action decisions to move can be explained by accumulation of activity to a motor threshold^53^, and response inhibition processes can similarly be modelled by drift diffusion models of activity accumulation for release or inhibition of an action^54,55^. Poorer interoceptive awareness could engender noisier afferent input to motor decision processes for homeostatic adjustment of behaviour, tipping accumulators for execution of action towards threshold. Further evidence for this putative mechanism comes from the association between faster Choose-Go reaction times and poorer interoceptive insight (high accuracy-confidence discrepancies), with potentially faster accumulator processes driving rapid ramps to action execution threshold in those with noisier interoceptive insight. However, the interoceptive insight discrepancy index is not a linear metric: a score of 0 indicates high interoceptive metacognition, with optimum alignment of accuracy and confidence. The further the discrepancy score from 0, the poorer the interoceptive metacognition, with negative discrepancy scores indicating low performance but high confidence, and positive discrepancy scores indicating high performance but low confidence. In our sample, five individuals who showed poorer insight, with low accuracy but high confidence, appear to behave similarly, in terms of faster reaction times, to those who also show poorer insight, but due to high accuracy and low confidence (Fig. 4c). This raises the possibility that insight abilities follow a ‘U-shaped’ curve, although in our present dataset, we were limited in our ability to explore this by the much smaller group of participants (n = 5) who showed low accuracy and high confidence, combined with the fact that the magnitude of their discrepancy is much smaller (0 to 0.3) than those showing the opposite (up to 0.7).

We did not observe effects between decisions to act and trait-level awareness of interoceptive ability, measured using trait interoceptive prediction error (TIPE), which indexes discrepancy between an individual’s objective (*accuracy*) and belief-based subjective (*sensibility*) dimensions of interoception^28,29^. State-based (e.g. *awareness* and *insight*) indices versus trait-based (TIPE) indices may reflect separate constructs neurally, and cognitively^56,57^.

In addition, it is notable that there were weak correlations between both intentional inhibition rates and Choose-Go reaction times with confidence ratings on the heartbeat tracking task, suggesting that the associations with the metacognitive interoceptive indices may be explained in part by cognitive processes that are unspecific to interoception. However, the larger Bayes Factors for the awareness and insight correlations than for confidence ratings hints that there is still a role for metacognitive interoceptive processes specifically in driving voluntary action, beyond generic judgements of mere confidence.

Interestingly, in people with Tourette Syndrome, who experience uncomfortable bodily sensations preceding involuntary tics, sensitivity to bodily sensations predicts severity of their premonitory urges to move^29,58^. This is further evidence that interoceptive signals can cue motor behaviour, via signals to midline motor regions^12,59^.

### Autonomic reactivity and trait impulsivity

We predicted that autonomic reactivity, as measured by heart rate variability, and trait impulsivity, measured by self-report questionnaires, might also shape reactive and intentional inhibition, as measured by NoGo and Choose-NoGo trials. Interestingly, despite previous associations between heart rate variability and inhibitory control^31–33^, we found no evidence for such a relationship within our data, including when additionally controlling for the potential confound of heart rate. The associations between autonomic reactivity and inhibitory control processes may occur more commonly under explicitly affective conditions, such as in the regulation of emotional expression^31^ or perhaps stopping in response to valenced stimuli^32^.

We did observe correlations between reactive inhibition (NoGo Errors), and intentional inhibition (% Choose-Go), with positive and negative urgency scores on the UPPS-P questionnaire. Urgency as measured by this questionnaire reflects a tendency to act impulsively while experiencing emotions (for example, “When I feel bad, I will often do things I later regret in order to make myself feel better now”). This perhaps lends weight to the conclusion that trait affective processes, which incorporate higher-order representation of the body, impact on frequency of volitional decisions to move or to inhibit. However, the Bayes Factors associated with this correlation was merely anecdotal in favour of H_1_. Otherwise, it is notable that other facets of trait impulsivity did not relate to intentional inhibition (or reactive): this may reflect a limitation of our (high-functioning) university student sample, in whom there are likely to be fewer individuals with very high (maladaptive) impulsivity levels than in the general population, and clinical samples may show otherwise.

We also tested whether trait impulsivity related to individual differences in interoception. Previously, we found a (non-significant) weak correlation between higher trait impulsivity on the BIS and poorer interoceptive accuracy on the heartbeat tracking task^18^, and that poorer performance on a two alternative forced choice synchronicity task predicted lower attentional impulsivity as measured by the BIS^60^. Here, having studied only the heartbeat tracking task, it was not possible to replicate Herman *et al.*^60^. Although we did not observe evidence for associations between heartbeat tracking accuracy and trait impulsivity in the present sample, there were positive correlations between BIS subscales and interoceptive awareness and confidence ratings. The anecdotal status of the Bayes Factors for these tests signifies the probably weak associations between interoceptive indices and trait impulsivity. This suggests that interoception may be a more potent driver of momentary urges to act, as measured by the intentional inhibition task, than trait-level endophenotypes; or alternatively, that such associations only become strongly evident in clinical populations.

### Study limitations and future directions

The heartbeat tracking task provides a valuable heuristic approach for quantifying individual differences in bodily sensitivity^41^. However, the task is subject to a number of confounds which limit its interpretation^61^. For example, participants may count elapsed seconds and use this as a proxy for number of heartbeats. Asking participants to estimate passage of time alongside estimation of heartbeats can provide a control^62,63^. Nevertheless, such task confounds may arguably influence our measures of metacognitive interoceptive awareness and insight, with which we observed an effect with intentional inhibition, to a lesser degree than objective task performance measures of interoceptive accuracy. Alternatively, two alternative forced choice tasks, in which participants report whether exteroceptive stimuli are synchronous or asynchronous with their heartbeat, can be employed^64^, but often many participants perform at chance, and so such approaches may not comprehensively capture individual differences in physiological and interoceptive function^61,63^. Recently developed heartbeat matching tasks, in which participants use a slider to adjust the rate of a pulsing visual stimulus to their own heart rate, show promise for addressing these methodological drawbacks^63^.

Interestingly, we did not replicate previously identified effects of cardiac cycle on reaction times^65,66^, whether for Go or Choose-Go trials. However, such previously reported effects have sometimes been small, and not present in both men and women^65^. In our recent study of reactive inhibition, Go trials during a stop signal task were no different in response time between systole and diastole^18^.

In both our present and our previous stop signal studies, we targeted the presentation of motor task cues according to timepoints when arterial baroreceptors – which underpin the neural and cognitive perception of a heartbeat – will be maximally active versus quiescent. Alternatively, one can study each individual participant’s ECG post-hoc to either pinpoint whether stimuli fell into particular cardiac phases^19^, or bin trials into histogram plots^21^. It may be important to bear in mind such different methodological approaches in timing task events to specific points within the cardiac cycle when comparing cardiac timing effects across studies.

Following completion of the intentional inhibition task, we recorded ECG for 2.5 minutes during which participants rested quietly with eyes open, to investigate HRV. Longer ECG recordings give more stable HRV estimates; however, a minimum of 1 minute is considered sufficient by consensus guidelines for reliable estimates of RMSSD^67^.

A complementary literature has examined the effect of motor acts on heart rate, leading to acceleration or deceleration during execution and inhibition of movements, respectively^23,25,68^. This includes both reactive inhibition^23^ and intentional inhibition on the ‘marble’ task^25^. Here, our focus was instead on the impact that cardiac cues have on motor behaviour: by dynamically monitoring the ECG signal, we ensured the timing of our stimulus delivery was accurate (Fig. 2), even taking into account momentary increases and decreases in heart rate. It is notable that reactive inhibition shows bidirectional effects between cardiac cycle and stopping^18,23^, while intentional inhibition effects appear to be unidirectional: with no evidence here for an effect of cardiac cycle on frequency of choosing to inhibit, but with cardiac deceleration occurring during intentional inhibition trials on the ‘marble’ task^25^.

To further examine how perception of the body and volitional urges to act or to withhold movements inter-relate, clinical samples of patient groups with altered interoception and motor function will be valuable. Both people with Tourette Syndrome and patients with Parkinson’s Disease show altered interoceptive processes, which predict premonitory urge and motor symptom severity^29,58,69^. Interestingly, however, patients with Parkinson’s Disease show equivalent intentional inhibition to controls on the ‘marble’ task^70^. Intentional inhibition, and its potential impact on volitional tic suppression, is yet to be fully explored in Tourette Syndrome.

## Conclusions

State interoceptive cues, namely heartbeats, do not influence voluntary decisions to act or withhold movement in an intentional inhibition task. However, higher-order perception of the body, according to interoceptive awareness and insight, is associated with individual differences in intentional inhibition rates and response times. Individuals with poorer metacognitive awareness of, and insight into, their interoceptive signals tend to move more and faster, while those with better awareness and insight tend to move less, and less rapidly. This suggests that noisier afferent input to motor decision processes may engender feelings of urges to move the body, which may be altered in clinical conditions such as Tourette Syndrome.

## Data Availability

The data reported in this study, related task code and analysis scripts, and .jasp statistical analysis file are available at https://osf.io/3xezy.
